# Maternal and placental microbiome and immune crosstalk in pregnancies with small-for-gestational-age fetuses – a pilot case-control study

**DOI:** 10.3389/fcimb.2025.1596588

**Published:** 2025-06-23

**Authors:** Katarzyna Kosińska-Kaczyńska, Dominika Krawczyk, Martyna Bednorz, Katarzyna Chaberek, Agnieszka Czapska, Magdalena Zgliczyńska, Krzysztof Goryca, Magdalena Piątkowska, Aneta Bałabas, Paweł Czarnowski, Natalia Żeber-Lubecka

**Affiliations:** ^1^ Department of Obstetrics, Perinatology and Neonatology, Centre of Postgraduate Medical Education, Warsaw, Poland; ^2^ Doctoral School of Translational Medicine, Centre of Postgraduate Medical Education, Warsaw, Poland; ^3^ Department of Genetics, Maria Sklodowska-Curie National Research Institute of Oncology, Warsaw, Poland; ^4^ Department of Gastroenterology, Hepatology and Clinical Oncology, Centre of Postgraduate Medical Education, Warsaw, Poland

**Keywords:** microbiome, pregnancy, fetal growth restriction, small-for-gestational-age, placenta

## Abstract

**Introduction:**

Pregnancies complicated by fetal growth restriction are associated with specific bacterial abundances and elevation of proinflammatory cytokines. The aim of the study was to simultaneously analyze the relation between the gut and placenta microbiome and cytokine profile in pregnant women with fetuses appropriate (AGA) and small for gestational age (SGA).

**Material and methods:**

Women with singleton pregnancies at or beyond 32 weeks of gestation were recruited. 11 delivered SGA newborns (study group) and 11 AGA newborns (control group). Samples of maternal venous blood, stool and placenta were collected perinatally.

**Results:**

In SGA group lower Chao index in placental samples collected from maternal side, while higher Chao index in placental samples collected from fetal side were observed. Taxonomic analysis identified four significantly less abundant genera in samples collected from maternal side. No taxa remained significant after correction in samples from fetal side, but several taxa showed trends of differing abundance. *Veillonella* showed a trend toward higher abundance in stool samples in SGA group, while other taxa were significant only at a lower threshold. Metabolite analysis revealed that hexanoic acid was significantly elevated compound in the stool of women from the SGA group. *Proteobacteria* unclassified and *Halomonadaceae* correlated with stool metabolites, while IL-6 and TNF-α correlated with specific bacterial groups.

**Conclusions:**

Specific changes in the gut microbiome and metabolome as well as placenta microbiome of women with SGA have been observed, with additional associations with inflammatory cytokine levels, suggesting a potential role of these factors in SGA development and highlighting the need for further research.

## 1 Introduction

The human intestine harbors a complex population of microorganisms that co-evolve with the host by maintaining a symbiotic relationship (Cheng et al., 2019). These microorganisms, including bacteria, fungi, and viruses, are present in numbers far exceeding those of host cells and are collectively referred to as the microbiota. The gut, vaginal, and oral microbiota undergo changes during pregnancy (Koren et al., 2024). The notion that the placenta harbors its own microbiome was first proposed by Aagaard et al. in 2014. The researchers, who studied placental samples after excision of maternal decidua and fetal chorion-amnion to avoid potential contamination, identified multiple phyla within the placental microbiome, namely *Firmicutes, Tenericutes, Proteobacteria, Bacteroidetes*, and *Fusobacteria* (Aagaard et al., 2014). Since then, numerous studies have either described the presence of a placental microbiome or argued against its existence (de Goffau et al., 2019; Theis et al., 2019; Williams et al., 2022; Stupak et al., 2023). Summarizing, Zakis et al. conducted a systematic review of studies investigating the existence and composition of the placental microbiome in healthy pregnancies (Zakis et al., 2022). They concluded that the placenta might be characterized by low microbial biomass, and that some of the microbial taxa identified in placental tissue may originate from the maternal oral cavity. However, they also noted that many of the microorganisms reported in placental samples were also detected in negative controls, suggesting that their presence may be attributable to contamination (Zakis et al., 2022). The authors of this review carefully assessed the risk of bias and selected few high-quality studies (Zakis et al., 2022). Among these, for instance, Lager et al. found no evidence of eukaryotic organisms in the placentas of either patients with adverse pregnancy outcomes or healthy controls (Lager et al., 2018). In contrast, Seferovic et al., based on their own research, concluded that the placenta is characterized by low abundance, low biomass, and sparse microbial populations and that its taxonomic composition is distinct from that observed in contamination controls (Seferovic et al., 2019). On the other hand, De Goffau et al. demonstrated that most bacterial deoxyribonucleic acid (DNA) detected in placental samples likely originates from contaminants, a conclusion also supported by Theis et al (de Goffau et al., 2018; Theis et al., 2019). It is important to highlight that studies on the placental microbiome often differ in their sampling techniques, which are critical for accurate interpretation of results. While some researchers, such as Seferovic et al., provide a detailed description of their methodology—including the removal of membranes from the fetal side —many studies lack such methodological transparency (Seferovic et al., 2019; Zakis et al., 2022). Thus, the question of whether the placenta possesses its own microbiome remains unresolved. For many decades, however, the uterus was also believed to be a sterile environment. Several studies have investigated the putative endometrial microbiota using 16S rRNA sequencing, each documenting the presence of a uterine microbiome (Franasiak et al., 2016; Moreno et al., 2016; Winters et al., 2019). Therefore, it is plausible that the placenta also harbors a unique microbiome on its maternal surface, as it is co-formed by the decidua.

A fetus classified as small-for-gestational-age (SGA) is typically defined as having an estimated weight below the 10^th^ centile for gestational age, while fetal growth restriction (FGR) refers to a condition in which the fetus is unable to reach its genetic growth potential (Lees et al., 2020). In pregnancies complicated by FGR, increased beta diversity, with a particular abundance of bacteria from the genera *Bacteroides, Faecalibacterium*, and *Lachnospira*, has been previously described (Tu et al., 2022). However, the maternal gut microbiota profile in pregnancies affected by FGR has rarely been reported. Abnormalities in the maternal cytokine profile have also been observed in women with FGR. Raghupathy et al. found elevated levels of interleukin 8 (IL-8) and reduced levels of IL-13 in the blood of women with FGR. Additionally, levels of IL-8, interferon γ (IFNγ), and tumor necrosis factor α (TNFα) were increased, while IL-10 levels were lower in women with FGR and placental insufficiency (Raghupathy et al., 2012). A dominance of pro-inflammatory cytokines has also been observed in other studies (Bartha et al., 2003; Al-Azemi et al., 2017).

The aim of the study was to analyze the relationship between the gut and placental microbiome and the cytokine profile of pregnant women with appropriate-for-gestational-age (AGA) and SGA fetuses.

## 2 Material and methods

This pilot study investigated the maternal microbiome and its relationship with the cytokine profile in pregnancies complicated by SGA. The study was funded by the National Science Centre in Poland (grant no. 2022/06/X/NZ5/01127).

### 2.1 Study population

Women with singleton pregnancies hospitalized at the Department of Obstetrics, Perinatology, and Neonatology at the Centre of Postgraduate Medical Education were recruited for participation. The inclusion criteria comprised maternal age of 18 years or older, singleton pregnancy, gestational age of 32 + 0 weeks or beyond, a viable fetus, verified gestational age, live birth, caesarean delivery with intact membranes and no uterine contractions, and informed consent provided by the participant. Birth weight centiles were estimated using the Fetal Medicine Foundation fetal and neonatal population weight charts (Nicolaides et al., 2018). Exclusion criteria included lack of informed consent, a history of intestinal surgery involving an intestinal stoma or bariatric surgery, immunosuppression, human immunodeficiency virus (HIV) infection or other conditions causing immune system dysfunction, intestinal dysbiosis syndrome, infectious diarrhea within the three months prior to enrolment, use of probiotics, antibiotics, or vaginal chemotherapeutics within the three months prior to enrolment, use of any vaginal medications within the same period, severe chronic diseases (renal failure, heart failure, liver failure, diabetes, or non-specific bowel disease), absence of an ultrasound scan performed between 11 and 14 weeks of gestation, genetic or major anatomical abnormalities in the fetus, known intrauterine infections leading to fetal growth restriction, and preterm rupture of membranes. Gestational age was calculated based on the first day of the last menstrual period or, in the case of assisted reproductive techniques, the day of embryo transfer, and was verified by crown-rump length measurement during the first-trimester ultrasound. Women who gave birth to SGA newborns were included in the study group, while those with AGA newborns formed the control group.

### 2.2 Stool samples

Stool samples were collected from each participant after recruitment into the study, during the third trimester of gestation and within seven days before delivery. Participants collected the samples themselves in sterile containers after receiving detailed instructions on the collection technique. The samples were then frozen at -20°C. Within one hour before delivery, 10 mL venous blood samples were collected from all enrolled women into polystyrene tubes containing tripotassium versenate (K3-EDTA). The blood samples were centrifuged at 1500 G for 10 minutes, 30 minutes after collection. The plasma was then frozen at -80°C. Once the study group was complete, the plasma samples were thawed, and cytokine concentrations were measured using the ELISA method. Tests were performed using standardized kits by BioLegend (San Diego, United States): IL6 - LEGENDplex™ Human IL-6 Capture Bead B3; IL8 - LEGENDplex™ Human CXCL8 (IL-8) Capture Bead B7; IL10 - LEGENDplex™ Human IL-10 Capture Bead B4; IFNγ, - LEGENDplex™ Human IFN-γ Capture Bead B5; TNFα - LEGENDplex™ Human TNF-α Capture Bead A8; CXCL12 - LEGENDplex™ Human CXCL12 (SDF-1) Capture Bead B6; CCL2 - LEGENDplex™ Human CCL2 (MCP-1) Capture Bead A10. The tests were performed in accordance with the manufacturer’s instructions.

### 2.3 Placenta samples

During the caesarean section, placental samples from both the maternal and fetal sides were collected immediately after delivery under sterile conditions. Immediately after placental delivery, under sterile conditions on the operating table, tissue samples were collected from the placenta. From the fetal side, approximately 10 g of tissue was obtained from the central part, while from the maternal side, approximately 10 g of tissue containing placental tissue and fetal membranes was collected eccentrically from the umbilical cord insertion site. The samples were then placed in sterile containers, sealed, and frozen at -20°C.

### 2.4 Placenta microbiome analysis

Bacterial genomic DNA was extracted from collected placental tissues using the QIAamp DNA Mini Kit (Qiagen, Germany). The bacterial 16S rRNA gene libraries were then prepared using the Ion 16S™ Metagenomics Kit and the Ion Plus Fragment Library Kit (Thermo Fisher Scientific, USA). Sequencing of the libraries (V2-4–8 and V3-6,7–9 regions) was performed on the Personal Genome Machine (PGM) platform (Thermo Fisher Scientific, USA) utilizing Ion Torrent technology and PGM™ Hi-Q™ View Sequencing Kit reagents (Thermo Fisher Scientific, USA), following previously established protocols (Zeber-Lubecka et al., 2024a). To minimize the risk of contamination - a major concern in placental microbiome studies - we implemented strict precautions throughout sample collection, processing, and analysis. Placental tissues were collected using sterile, DNA-free instruments, with personnel wearing single-use gloves and following aseptic techniques. All samples were handled in a laminar flow hood decontaminated with bleach and UV irradiation. DNA extraction protocols included multiple negative controls, such as blank extraction and no-template PCR controls. Environmental and reagent controls were also sequenced to monitor potential background microbial signals. Bioinformatic analyses incorporated contamination-aware tools to distinguish true microbial signal from potential contaminants. These measures collectively ensured the integrity of the microbiome profiles analyzed in this study.

### 2.4 Fecal microbiome and metabolome analysis

DNA was extracted from fecal samples using the QIAamp Fast DNA Stool Mini Kit (Qiagen, Hilden, Germany) following the manufacturer’s protocol. The extracted DNA was quantified using a Qubit dsDNA High Sensitivity Assay kit via fluorimetry (Thermo Fisher Scientific, Carlsbad, CA, USA). Metagenomic sequencing was performed on the Illumina NovaSeq 6000 platform (San Diego, CA, USA) with 10 ng of DNA input. The sequencing utilized 100-base pair paired-end reads, adhering to the standard guidelines provided by the manufacturer (Zeber-Lubecka et al., 2024b). Metabolites, short chain fatty acids (SCFAs) and amino acids (AAs), were isolated from frozen stool samples, chemically derivatized, and analyzed through gas chromatography. The analysis was conducted using an Agilent 7000D Triple Quadrupole mass spectrometer integrated with a 7890 GC System and a G4513A autosampler (Agilent Technologies, Santa Clara, CA, USA), following established protocols (Kulecka et al., 2022; Kulecka et al., 2023).

### 2.5 Statistical analysis

Alpha diversity metrics were calculated using the iNEXT package (version 3.0), including the Shannon index, which accounts for both species abundance and evenness under the assumption of random sampling, as well as the Chao index, which estimates species richness by emphasizing the presence of rare taxa. For amplicons taxa were identified with Mothur (version 1.48) using Silva database (version 132). Statistical comparisons were performed using the Kruskal–Wallis test or the Mann–Whitney U-test for analyses involving only two groups. Bacterial taxa were identified with Kraken2 (version 2.1.3) using its default settings and databases. Species-level classifications were achieved with Bracken (version 2.7), applying a minimum threshold of 100 counts. Variations in taxa abundance between groups were analyzed using the LINDA method [Linear (Lin) Model for Differential Abundance (DA)] (Zhou et al., 2022), tailored for compositional data. The Mann-Whitney U test was used to compare the ratio of Bacteroidetes to Firmicutes (F/B ratio). P-values were adjusted using the Benjamini–Hochberg (Benjamini and Hochberg, 1995) procedure to control the false discovery rate (FDR). For metabolite concentration differences between groups, the Mann–Whitney U-test was utilized. Correlation between taxa and metabolites was tested using Spearman’s correlation coefficient. Taxa clearly linked to at least one metabolite were identified using the metadeconfoundR package. Only taxa with an average of more than 1000 assigned reads and present in at least 10% of the samples were included in the analysis. Regularized Canonical Correlation Analysis was conducted on these taxa and their associated metabolites using the Ridge method, with parameter tuning following the mixOmics tutorial guidelines (rCCA nutrimouse case study|mixOmics). The correlation structure was illustrated using the complexHeatmap package. Bacterial species were grouped using Ward’s clustering method (“ward.D2” method via the base R hclust function). The dynamicTreeCut package was employed to determine the optimal number of modules. Taxon Set Enrichment Analysis (TSEA) was performed using MicrobiomeAnalyst to identify biologically meaningful patterns among functionally related stool microbial groups, allowing detection of subtle but consistent changes without relying on arbitrary significance thresholds. Statistical analysis of study participants characteristics as well as comparison of the results of laboratory examinations was performed using STATISTICA 13 software (TIBCO Software Inc.). Nonparametric tests were used for comparisons - for two independent groups the Mann-Whitney U test, while Spearman’s R was used to assess the correlation between two variables. A p value of <0.05 was considered statistically significant.

The study protocol was approved by the Ethics Committee at the Centre of Postgraduate Medical Education (approval no. 46/2022) and was conducted in accordance with the Declaration of Helsinki.

## 3 Results

### 3.1 Characteristic of the study group

A total of 22 women were included in the study, with 11 in the SGA group and 11 in the AGA group serving as the control. The study sample size was determined by the funding received. The basic characteristics of the study participants are presented in **Table 1**. There were no statistically significant differences between the analyzed groups, except for the newborns’ birth weights, which were significantly lower in the SGA group.

**Table 1:** Basic characteristics of the study groups.

Characteristics	Study group N=22 median (interquartile range)*	SGA group N=11 median (interquartile range)*	AGA group N=11 median (interquartile range)*	p-value
Age (years)	31.5 (26-39)	31 (26-38)	32 (27-39)	0.8
Primiparous**	13 (59.1)	8 (72.7)	5 (45.5)	0.4
Gestational age at stool sample collection (weeks)	37 (36-38)	36 (36-37)	38 (36-40)	0.6
Hypertension**	1 (4.5)	0	1 (9.1)	1
Preeclampsia**	2 (9.1)	2 (18.2)	0	0.5
Gestational age at delivery (weeks)	37.5 (36-40)	37 (36-38)	38 (37-40)	0.7
Newborn’s birthweight (g)	2635 (1940-3660)	2080 (1615-2180)	3320 (3080-3860)	0.01
Apgar score <= 7 points**	1 (4.5)	1 (9.1)	0	1

*unless otherwise stated; **- number (%); SGA, small-for-gestational-age newborns group; AGA, appropriate-for-gestational-age newborns group.

### 3.2 Maternal venous blood cytokine profile

The cytokine concentration values measured in maternal venous blood are presented in **Table 2**. No significant differences were observed between the groups.

**Table 2:** Blood cytokine profile in SGA and AGA groups.

Cytokine	SGA group N=11 median (interquartile range)	AGA group N=11 median (interquartile range)	p-value
IL-6 (pg/mL)	1.638 (1.638-2.399)	1.638 (1.638-5.443)	0.6
IL-8 (pg/mL)	1.78 (1.78-2.875)	1.78 (1.78-7.2550	0.9
IL-10 (pg/mL)	1 (1-2.094)	1 (1-1.875)	0.6
IFNγ (pg/mL)	1.461 (0.184-2.738)	1.461 (0.576-2.738)	0.8
TNFα (pg/mL)	0.01 (0.001-0.05)	0.01 (0.001-0.06)	0.3
CXCL12 (pg/mL)	2269.145 (1427.313-2515.263)	1560.345 (1164.841-2080.619)	0.2
CCL2 (pg/mL)	167.208 (121.809-217.699)	155.615 (100.890-225.945)	0.5

SGA, small-for-gestational-age newborns group; AGA, appropriate-for-gestational-age newborns group.

### 3.3 Quality control of sequencing data

After sequencing of the maternal and newborn placenta samples and maternal stool samples all reads were monitored for quality control purposes. The criteria for quality assessment are outlined in the Methods section provided above. A total of (Kenfack-Zanguim et al., 2023) samples produced sequences matching the quality criteria. Sequencing of the microbial DNA collected by the maternal and newborn placenta samples resulted in (10,5M) reads of which (8,2M) reads passed quality control and could be assigned to a taxon. In maternal stool samples the values included (1139M, 690M), respectively. Maternal and newborn placenta and stool samples yielded a median of 145,000 and 50 million reads per sample, respectively. A total of 1,374 species and 539 genera were detected in the placental samples, with an average of 270 assigned species and 129 genera per sample. Among these, 86 species reached at least 1% relative abundance in at least one sample (**Supplementary Table 1**).

### 3.4 Stool samples diversity, bacterial abundance and stool metabolites levels

**Figure 1:**
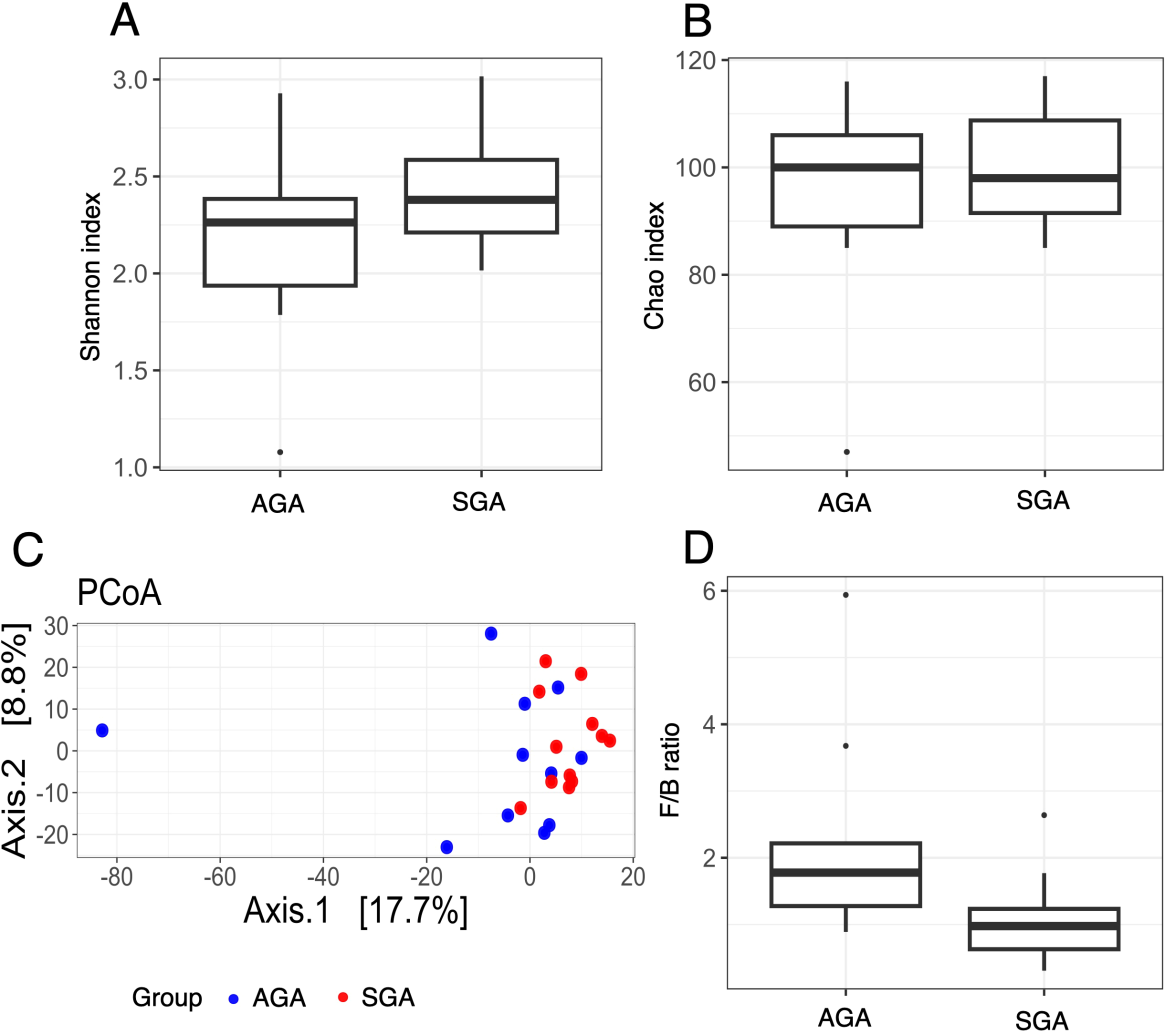
Bacterial diversity in stool samples measured with the **(A)** Shannon, **(B)** Chao, **(C)** PCoA and **(D)** F/B ratio in the SGA and AGA groups.

An analysis of the stool sample diversity was conducted to compare mothers in the test and control groups. Alpha diversity measures, including Shannon (**Figure 1A**) and Chao (**Figure 1B**) indices, showed no statistically significant differences between the groups. However, beta diversity analysis revealed a significant difference in the first principal coordinate between the groups, as indicated by the Wilcoxon test (p=0.0086). No significant difference was observed in the second principal coordinate (p=0.4865) (**Figure 1C**). A significant difference in the F/B ratio was observed between the SGA and control groups, with a lower value in the SGA group (p adj=0.03) (**Figure 1D**). At the genus level, only *Veillonella* showed a trend towards differentiating maternal stool samples from the SGA and AGA groups (p-adjusted =0.06), with higher abundance observed in the SGA samples. The abundances of other taxa, including *Limosilactobacillus, Bacteroides, Arabiibacter*, and *Oxalobacter*, were statistically significant only at p <0.05 (**Table 3**).

**Table 3:** Bacteria at the genus level differentiating maternal stool samples of SGA and AGA groups.

Taxa	baseMean	log2FoldChange	p-value	Adjusted p-value
***Veillonella***	0.40	**8.24**	0.000394	0.061
***Limosilactobacillus***	0.07	**6.76**	0.002486	0.192
***Bacteroides***	260086.80	**2.27**	0.004603	0.237
***Arabiibacter***	0.07	**3.26**	0.006732	0.257
***Flavonifractor***	5640.88	**1.39**	0.008781	0.257
***Schaalia***	86.91	**1.64**	0.009968	0.257
***Anaerostipes***	668.44	**1.90**	0.020779	0.311
***Bifidobacterium***	11786.90	**2.10**	0.022	0.311
***Intestinibaculum***	0.12	**3.43**	0.023	0.311
***Lughvirus***	0.13	**4.11**	0.024	0.311
***Enterobacter***	0.074067	**4.78**	0.025	0.311
***Phocaeicola***	58145.05	**2.45**	0.025	0.311
***Butyricicoccus***	0.19	**3.92**	0.029	0.311
***Actinomyces***	1.19	**4.37**	0.029	0.311
***Rothia***	2.28	**4.79**	0.030	0.311
***Granulicatella***	0.07	**3.38**	0.037	0.348
***Lactiplantibacillus***	0.07	**4.01**	0.038	0.348
*Oxalobacter*	46.54	-5.34	0.045	0.355

Bolded – bacteria more abundant in the stool samples of tested patients at p value <0.05.

Functional enrichment analysis using MicrobiomeAnalyst was performed based on bacterial taxa identified in stool samples, aiming to explore potential metabolic pathways associated with the observed microbial composition. The analysis was performed at the Mixed-Level Taxon resolution using taxon set libraries available in MicrobiomeAnalyst, including Host–Diet, Microbiome-Intrinsic, and Host-Intrinsic taxon sets. Within the Host–Diet taxon set enrichment analysis; several microbial patterns were significantly associated with dietary contexts. Notably, taxa linked to *arabinoxylan oligosaccharides and obesity* showed the strongest enrichment (p adj = 2.43 × 10^−1^), followed by sets associated with *high-fat diet* and *omega-3 intake and health* (p adj = 0.00334 for both), suggesting potential dietary–microbiota interactions differing between the study and control groups. Analysis of Microbiome-Intrinsic taxon sets revealed trends toward enrichment of butyrate-producing bacteria (raw p = 0.022), although this did not reach statistical significance after multiple testing correction (adjusted p = 1.0) (**Supplementary Table 2**).

**Figure 2:**
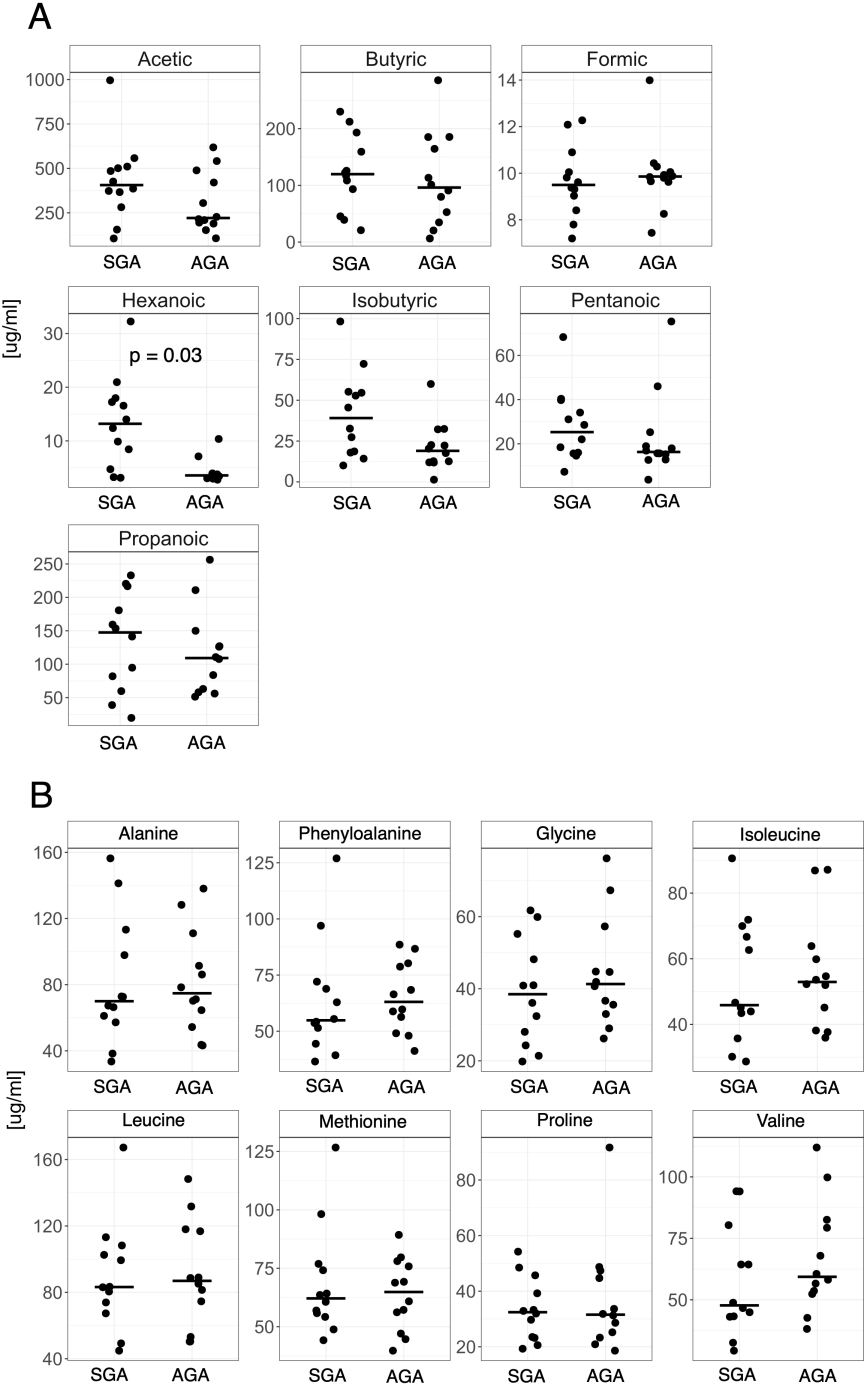
The relative fecal concentrations of **(A)** short-chain fatty acids (SCFAs) and **(B)** amino acids (AAs) in SGA compared to AGA group.

Next, we used GC/MS-based analysis of fecal samples to identify seven SCFAs and eight AAs. We compared the relative concentrations of metabolites per gram of stool weight (**Figure 2**) in the maternal stool samples. Only one metabolite, hexanoic acid, was statistically significant (p = 0.03), and its concentration was higher in stool samples from women in the SGA group (**Figure 2A**).

### 3.5 16S rRNA-based profiling of the maternal and fetal placental microbiota

**Figure 3:**
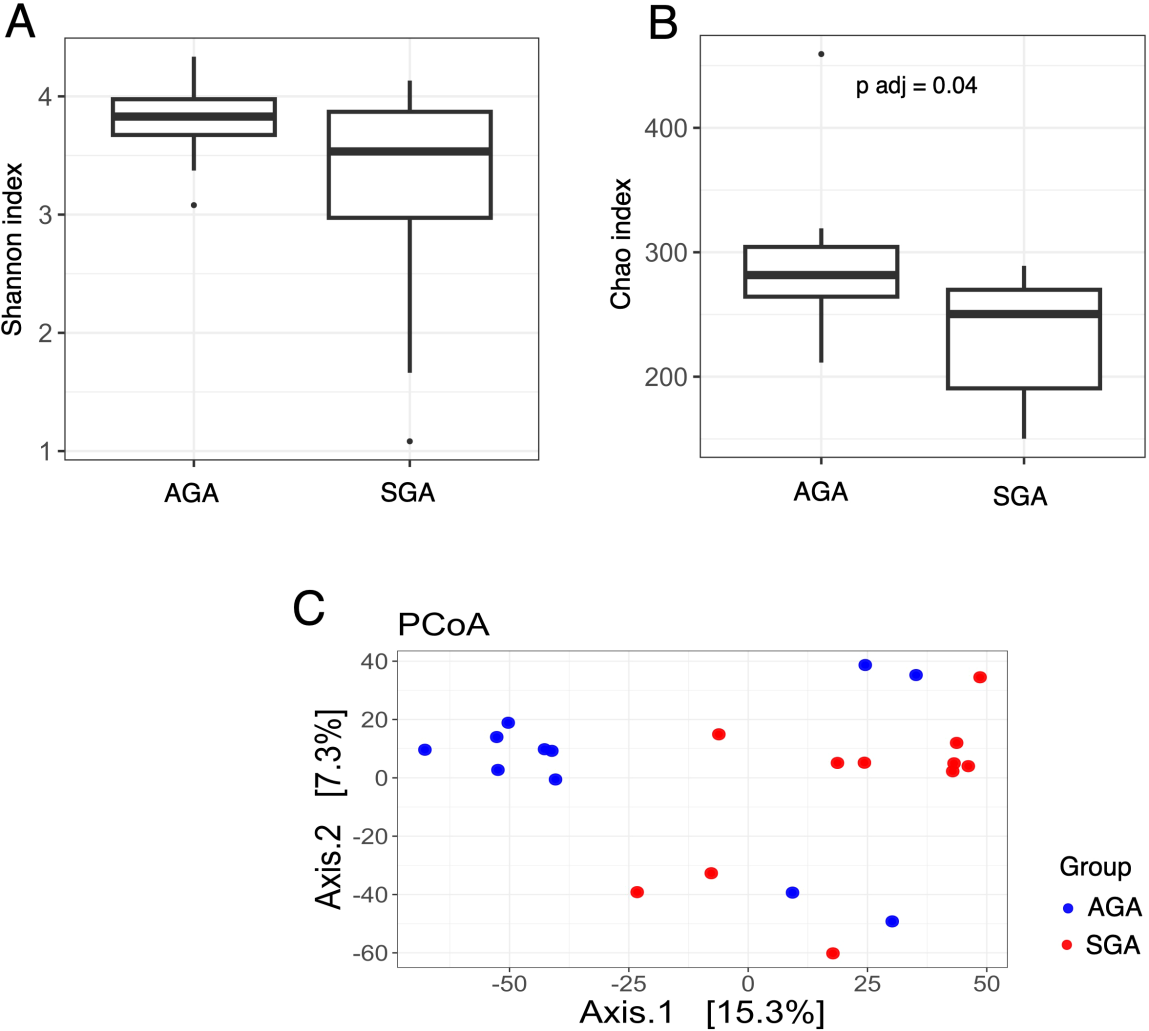
Bacterial diversity measured with the **(A)** Shannon, **(B)** Chao, **(C)** PCoA indices in the microbiota of placenta samples collected from the maternal side of the AGA and SGA groups.

**Figure 4:**
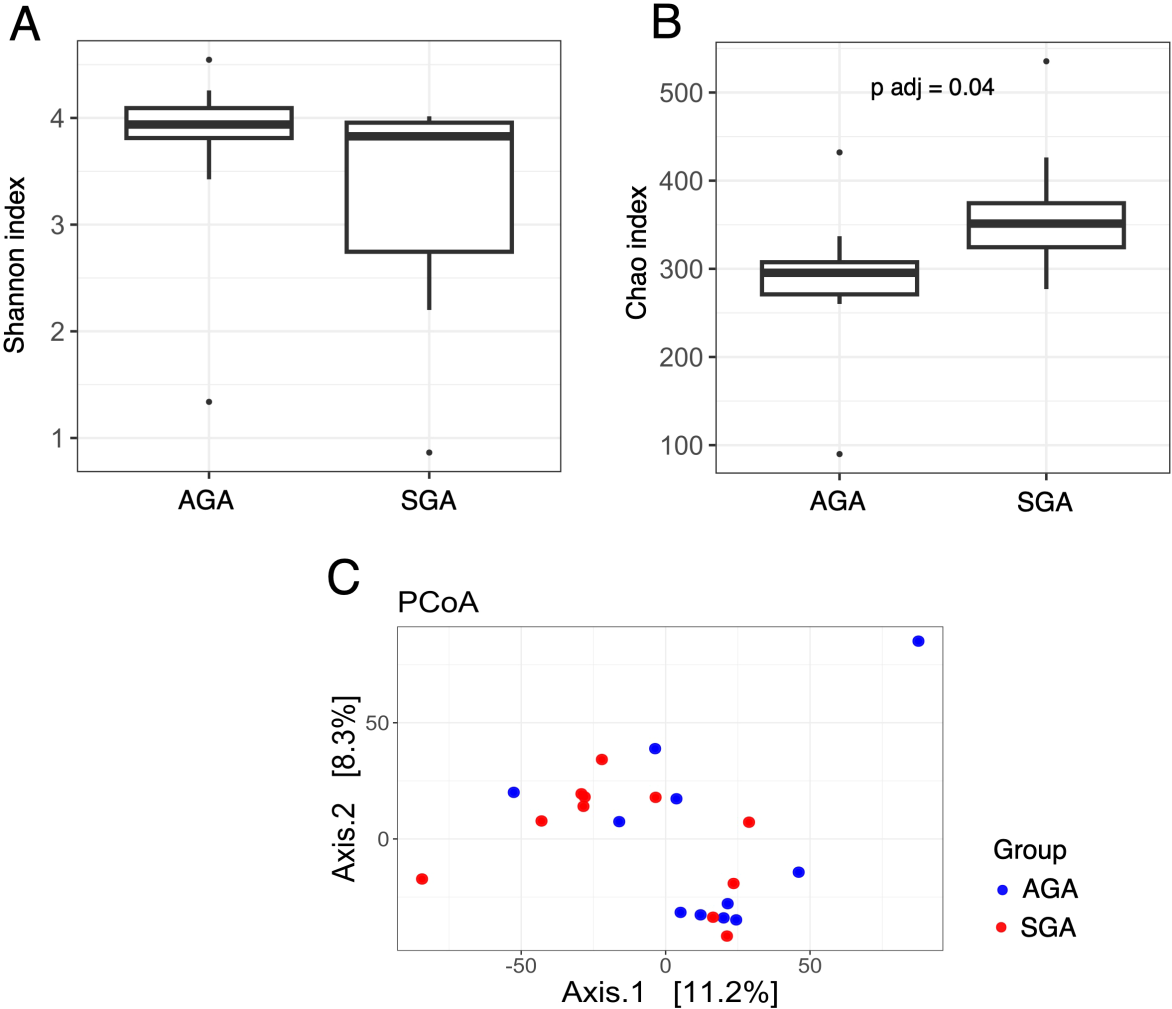
Bacterial diversity measured with the **(A)** Shannon, **(B)** Chao, **(C)** PCoA indices in the microbiota of placenta samples collected from the fetal side of the AGA and SGA groups.

The structure of the bacterial community among samples was evaluated by analyzing the α- and β-diversity at the genus level. The α-diversity was analyzed using the Shannon index, a marker of bacterial richness and evenness and Chao index for bacterial richness calculation. The β-diversity was analyzed using principal component analysis (PCoA). To visualize microbial composition and explore group-specific patterns, we compared taxonomic profiles of placental samples from SGA and AGA pregnancies, including both maternal and fetal sides. The distribution and relative abundance of dominant bacterial genera across placental samples, including comparisons by fetal growth status and placental side, are demonstrated in **Supplementary Figures 1** and **2**. We conducted analyses comparing placental samples from the maternal side of the SGA group with placental samples from the maternal side of the AGA group. We demonstrated that, after multiple hypothesis testing corrections, the Chao index (**Figure 3B**) was significantly lower in the maternal placentas of the SGA samples compared to the maternal placentas of the AGA group (adjusted p-value, p adj = 0.04), while the Shannon index remained unchanged (**Figure 3A**). Interestingly, analyses performed on placental samples from the fetal side in the SGA group compared to the AGA group revealed a statistically significant increase in the Chao index (**Figure 4B**) in the microbiota of the AGA samples (p adj = 0.04), with the Shannon index remaining unchanged (**Figure 4A**). No differences in beta diversity were observed between the studied groups (**Figures 3C**, **4C**).

**Figure 5:**
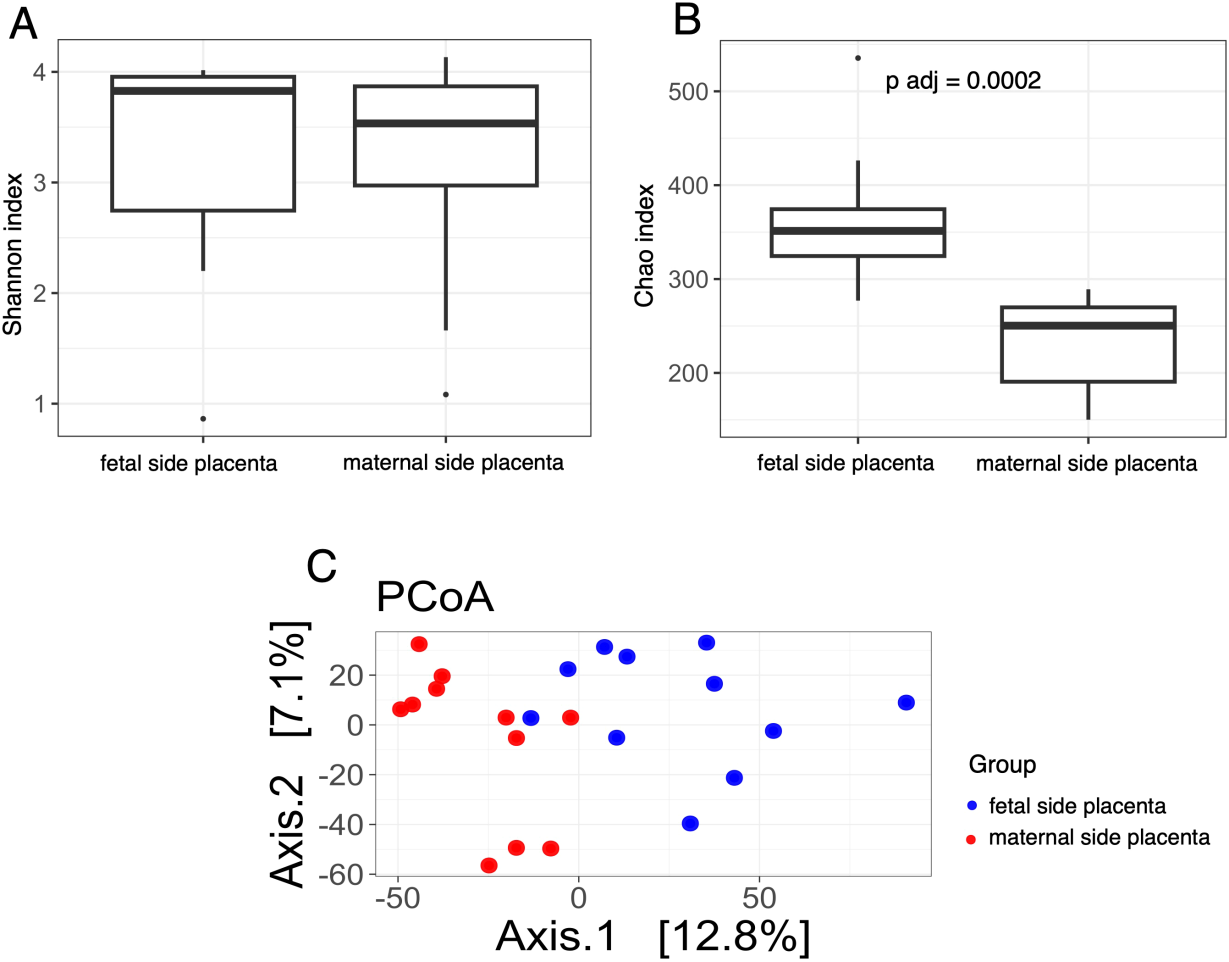
Bacterial diversity measured with the **(A)** Shannon, **(B)** Chao, **(C)** PCoA indices in the in the microbiota of placenta samples collected from the maternal and fetal side of the SGA group.

**Figure 6:**
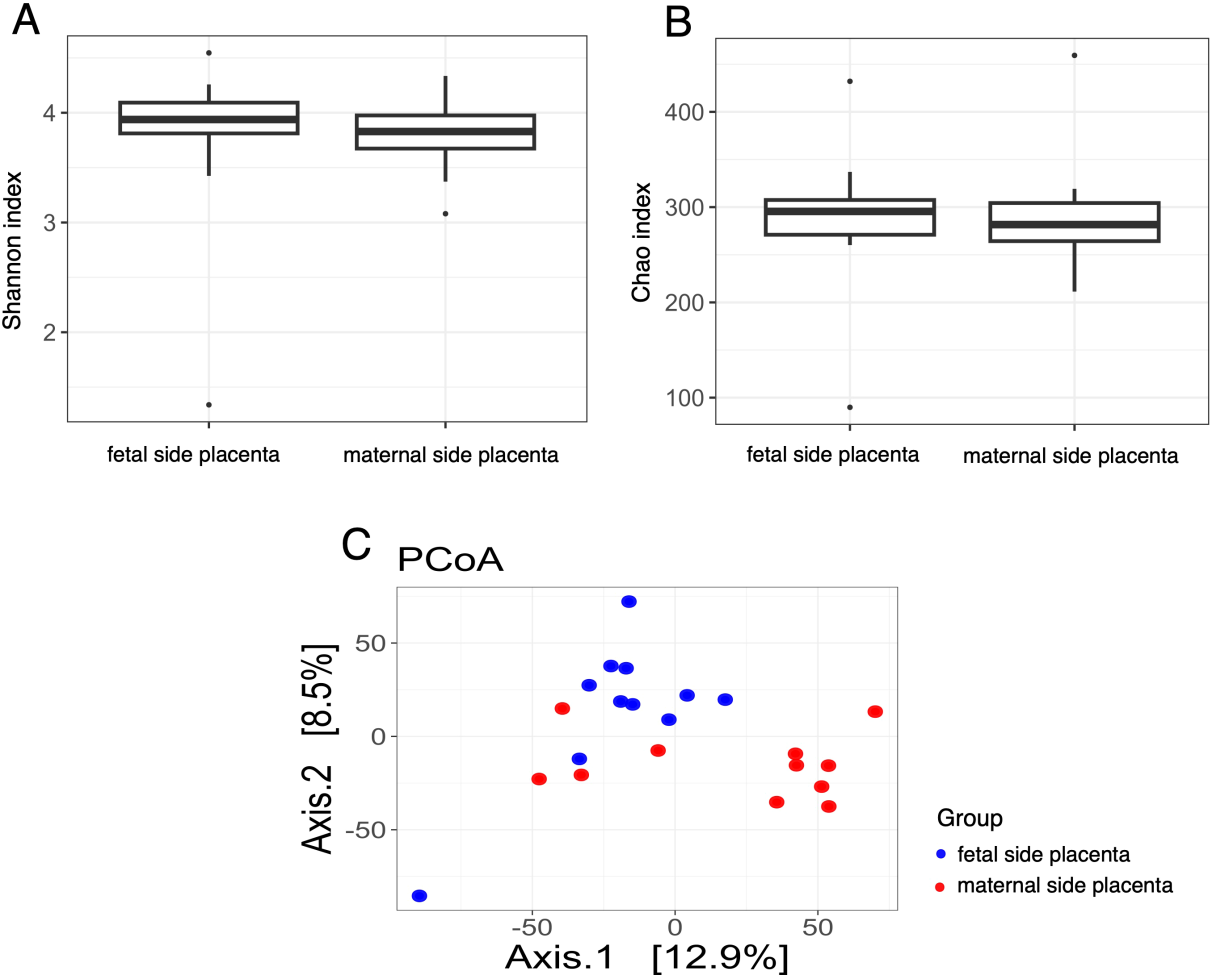
Bacterial diversity measured with the **(A)** Shannon, **(B)** Chao, **(C)** PCoA indices in the in the microbiota of placenta samples collected from the maternal and fetal side of the AGA group.

Subsequently a comparison of the microbiota of placental samples from the maternal side with samples from the fetal side in the SGA group was performed. The results showed a statistically significant higher Chao index in samples taken from the fetal side of the placentas in this group (p adj = 0.0002) (**Figure 5B**). In contrast, a similar comparison for the AGA group revealed no significant differences in the Chao index (**Figure 6B**). Furthermore, once again, the Shannon index values for both compared groups were not statistically significant (**Figures 5A** and **6A**). The results showed a highly significant difference between the placenta of the mother and the placenta of the fetus in terms of the first principal coordinate (PCoA_1), with p-values of 0.0001 (t-test) and 3.97E-05 (Wilcoxon test) in SGA group (**Figure 5C**). However, no significant difference was observed in the second principal coordinate (PCoA_2), suggesting that the main variation between these groups was captured in the first dimension. In turn, the results showed a statistically significant difference between the placenta of the mother and the placenta of the fetus in terms of the first and second principal coordinates (PCoA) in AGA group (**Figure 6C**). Specifically, the p-values from the t-test and Wilcoxon test suggested that the differences in PCoA_1 (p = 0.017) and PCoA_2 (p = 0.006) were unlikely to be due to random chance, supporting the hypothesis that these groups differed in microbial composition or another measured characteristic.

### 3.5 Taxonomic profiling

LINDA-based analyses demonstrated that, at the genus level, the abundances of four taxa differed significantly between placental samples from the maternal side in the SGA and AGA groups (p-adjusted <0.05). All four taxa - *Ruminiclostridium 6, Lachnospiraceae A2, Corynebacteriaceae*, and *Faecalibaculum* — were less abundant in placental samples from the SGA group (**Table 4**). For comparisons between placental samples from the fetal side in the SGA and AGA groups, no taxa exhibited significant differences after correction for multiple testing (p-adjusted <0.05). However, 26 taxa were observed at an uncorrected significance level of p <0.05, including 15 taxa that were more abundant and 11 that were less abundant in placental samples from the SGA group (**Supplementary Table 3**).

**Table 4:** Bacteria at the genus level differentiating placental samples collected from the maternal side of SGA and AGA groups.

Taxa	baseMean	log2FoldChange	p-value	Adjusted p-value
*Ruminiclostridium 6*	2361.36	-5.64	9.52E-05	0.033
*Lachnospiraceae A2*	1165.86	-6.37	0.000141	0.033
*Corynebacteriaceae*	45.520	-1.79	0.000345	0.054
*Faecalibaculum*	2213.26	-5.63	0.000486	0.057

We also identified differentiating taxa in the comparison of placental samples collected from the maternal and fetal side within the SGA group. Five taxa remained significant after correction for multiple testing (p-adjusted <0.05), while three additional taxa showed a trend towards significance (p-adjusted <0.1). All identified bacterial taxa were less abundant in placental samples from the fetal side of the SGA group (**Table 5**). In the same comparison of placental samples from the maternal and fetal side in the AGA group, all identified bacterial taxa were more abundant in placental samples collected from the fetal side (p-adjusted <0.05) (**Table 6**).

**Table 5:** Bacteria at the genus level differentiating placental samples collected from the maternal and fetal side in SGA group.

Taxa	baseMean	log2FoldChange	p-value	Adjusted p-value
***Chitinophagaceae unclassified***	2175.99	-7.30	1.23E-08	**6,8E-06**
***Sediminibacterium***	261.24	-4.60	5.36E-07	**0.000148**
***Rhodocyclaceae unclassified***	41864.06	-8.06	1.59E-06	**0.000292**
***Dechloromonas***	5549.48	-8.03	2.68E-06	**0.000369**
***Methyloversatilis***	972.08	-5.80	0.000229	**0.025**
*Rhodospirillales uncultured*	185.82	-4.10	0.000723	0.064
*Deinococcaceae unclassified*	875.44	-4.22	0.000824	0.064
*Tepidicella*	2584.36	-4.38	0.00145	0.099

Bolded – taxa significant after correction for multiple testing (adjusted p-value <0.05).

**Table 6:** Bacteria at the genus level differentiating placental samples collected from the maternal and fetal side in AGA group.

Taxa	baseMean	log2FoldChange	p-value	Adjusted p-value
***Bifidobacteriaceae unclassified***	323.94	4.12	1.64E-05	**0.0089**
***Erysipelotrichaceae unclassified***	12.05	3.00	4.02E-05	**0.011**
***Selenomonadales unclassified***	12.62	3.60	0.000167	**0.030**
***Ruminiclostridium 6***	80.85	5.60	0.000222	**0.030**
***Prevotellaceae uncultured***	27.71	5.66	0.000312	**0.030**
***Dorea***	29.97	5.58	0.00034	**0.030**
***Lachnospiraceae A2***	19.00	6.67	0.000387	**0.030**
*Acidaminococcus*	52.55	6.44	0.00084	0.057
*Corynebacteriaceae*	18.82	2.01	0.000977	0.059
*Erysipelotrichaceae uncultured*	10.91	3.89	0.00111	0.061

Bolded – taxa significant after correction for multiple testing (adjusted p-value <0.05).

### 3.7 Correlation between bacteria populations, stool metabolites and plasma cytokines

Six correlations (Spearman’s coefficients with an absolute value greater than 0.6) were identified between taxa abundance and various variables. The most abundant taxa correlated with stool pentanoic and butyric acids were *Proteobacteria* (unclassified), which showed an inverse correlation, and *Halomonadaceae*, which exhibited a positive correlation (**Table 7**). Plasma cytokines IL-6 and TNF-α positively correlated with *Xanthomonadales* (unclassified) and *Firmicutes* (unclassified genera), respectively. A summary of all taxa correlations with variables, including both statistically significant and non-significant results, is presented in **Supplementary Table 4**.

**Table 7:** Correlations between taxa abundance and variables.

Taxa	Variable	Spearman coefficient	p-value
*Proteobacteria unclassified*	Pentanoic	-0.67	0,0008
*Halomonadaceae*	Butyric	**0.66**	0,0008
*Xanthomonadales unclassified*	IL 6	**0.66**	0,0008
*Firmicutes unclassified*	TNF α	**0.69**	0,0004

The correlations presented are for Spearman coefficient with absolute value larger than 0.6. Positive correlations are bolded.

## 4 Discussion

This is the first study to investigate the maternal gut and placental microbiota and its relationship with the cytokine profile in women with SGA and AGA newborns. We observed a lower Chao index in placental samples collected from the maternal side, while a higher Chao index was observed in placental samples collected from the fetal side in SGA pregnancies. Taxonomic analysis identified four genera that were significantly less abundant in placental samples from the maternal side in the SGA group, whereas no taxa remained significant after correction on the fetal side. However, several taxa showed trends towards differing abundance. Stool sample analysis revealed no significant differences in α-diversity between the SGA and AGA groups based on the Shannon and Chao indices. However, β-diversity analysis indicated a significant difference in the first principal coordinate. Additionally, the F/B ratio was significantly lower in the SGA group compared to the AGA group. *Veillonella* showed a trend towards higher abundance in SGA stool samples, while other taxa were significant only at a lower threshold. Metabolite analysis identified hexanoic acid as the only significantly elevated compound in the stool of women from the SGA group. *Proteobacteria* (unclassified) and *Halomonadaceae* correlated with stool metabolites, while IL-6 and TNF-α correlated with specific bacterial groups.

The maternal gut microbiota plays a crucial role in metabolism, immunity, and nutrient absorption. Several studies have identified links between gut microbiota and fetal growth. Published data indicate differences in the gut microbiome between pregnant women with SGA and AGA fetal growth; however, findings are sometimes inconclusive. Tang et al. observed an increase in the microbial population, microbial richness, and community richness, alongside a decrease in microbial diversity in the maternal gut microbiome of women carrying growth-restricted fetuses. PCoA analysis showed both overlap and separation between the AGA and FGR groups. The researchers found that *Bacteroides, Akkermansia, Eubacterium coprostanoligenes* group, *Phascolarctobacterium, Parasutterella, Odoribacter, Lachnospiraceae UCG_010*, and *Dielma* were significantly more abundant in the FGR group, whereas *Dialister, Tyzzerella, Collinsella, Roseburia, Intestinibacter, Monoglobus, Clostridium sensu stricto 1, Veillonella, Corynebacterium, Anaerococcus, Staphylococcus, Eubacterium, DTU089*, and *Eubacterium brachy* group were significantly less abundant in the FGR group (Tang et al., 2024). He et al. identified significant differences in the abundance of 20 gut microbial taxa in the gut microbiome of mothers with FGR and AGA newborns. Their findings indicated a positive correlation between the genus *Roseomonas* and unclassified *Propionibacteriaceae*, while a negative correlation was observed between the genus *Marinisporobacter* and *Sphingomonas* and neonatal birthweight percentile (He et al., 2023). Tu et al. compared the gut microbiota of 14 pregnant women with SGA and 18 with AGA fetal growth using 16S rDNA amplicon sequencing. The authors identified significant differences in β-diversity between the groups. At the genus level*, Bacteroides, Faecalibacterium*, and *Lachnospira* were highly abundant in women from the SGA group (Tu et al., 2022). Similarly, according to Xiao et al., enriched bacterial operational taxonomic units (OTUs) of the genus *Bacteroides* were observed in the maternal gut microbiome of the FGR group (Xiao et al., 2024). A high abundance of *Bacteroides* from the phylum Bacteroidota was also noted by Tu et al. in the maternal gut microbiome of women with SGA fetal growth (Tu et al., 2022). *Bacteroides* in the intestines is associated with lipid metabolism and has been linked to maternal dyslipidaemia during pregnancy (Yang et al., 2023). Previous research has suggested that maternal dyslipidaemia may be associated with accelerated placental epigenetic ageing, which in turn can lead to placental insufficiency and pregnancy complications such as preeclampsia, preterm delivery, or SGA infants (Shrestha et al., 2019). Therefore, it is possible that an increased abundance of *Bacteroides* in the maternal gut contributes to the development and progression of FGR through lipid metabolic pathways.

Additionally, *Bacteroides, Faecalibacterium*, and *Lachnospira* are significantly enriched in Kyoto Encyclopedia of Genes and Genomes (KEGG) pathways related to glycometabolism (Tu et al., 2022), suggesting that this specific shift in the gut microbiome may also influence glucose metabolism. *Bacteroidetes* is a major contributor to lipopolysaccharide (LPS) biosynthesis in the intestines (Tu et al., 2022), and its high abundance may induce inflammation during pregnancy (Wang et al., 2020). Animal studies have shown that maternal LPS exposure in late gestation results in intrauterine FGR in mice (Xu et al., 2005; Zhao et al., 2013). The data on maternal gut microbiota in FGR pathology is, however, varied and sometimes contradictory. Tao et al. investigated a cohort of 35 women with FGR and 35 with AGA pregnancies. At the species level, the abundances of *Lactobacillus* and *Catenibacterium* were elevated in the SGA group, whereas the abundances of *Ruminococcaceae, Bacteroides uniformis, Mollicutes RF39*, and *Alistipes onderdonkii* were reduced in the gut microbiota of pregnant women carrying FGR fetuses. The researchers also observed that the abundance of *Catenibacterium* was negatively correlated, while *Lachnospiraceae* was positively correlated, with neonatal birth weight (Tao et al., 2023).

Altered gut microbiota may activate inflammatory pathways, leading to dysbiosis and increased intestinal permeability. Both bacteria and their metabolites can pass into the maternal circulation and either migrate to the placenta or influence its function (La et al., 2022; Lopez-Tello et al., 2022). Abnormal fetal growth is often associated with placental insufficiency and preeclampsia. Chen et al. conducted a case-control study comparing the gut microbiome of normotensive and preeclamptic women. The authors found reduced bacterial diversity in preeclamptic women, with an enrichment of opportunistic pathogens, particularly *Fusobacterium* and *Veillonella*, and a marked depletion of beneficial bacteria, including *Faecalibacterium* and *Akkermansia*. To investigate the causative relationship between gut dysbiosis and the development of preeclampsia, the researchers performed fecal microbiota transplantation in an antibiotic-treated mouse model. Animals transplanted with microbiota from preeclamptic donors exhibited lower fetal and placental weights (Chen et al., 2020).

During our study, we observed a positive correlation between plasma IL-6 and TNF-α and *Xanthomonadales* and *Firmicutes*. Similar associations have been reported in the literature. Orbe-Orihuela et al., in a study involving children with obesity, found a high relative abundance of *Firmicutes* correlated with increased levels of TNF-α (Orbe-Orihuela et al., 2018). Bahar-Tokman et al., investigating a population of patients with type 2 diabetes, reported an elevated *Firmicutes*-to-*Bacteroidetes* ratio, with *Firmicutes* positively correlating with the expression of pro-inflammatory cytokine genes (Bahar-Tokman et al., 2022). Although data on the *Xanthomonadales* are limited, previous study have demonstrated an association between the presence of *Xanthomonadales* in the vaginal microbiome and preterm prelabor rupture of membranes (Mu et al., 2023). Furthermore, the abundance of *Veillonella*, which we identified in the gut microbiota of pregnant women from SGA group, has previously been associated with pro-inflammatory dietary patterns, gastric cancer, and autoimmune hepatitis (Wei et al., 2020; Rocha et al., 2023; Nath and Natarajan, 2024).

The metabolomic composition in SGA pregnancies differs from that in AGA pregnancies. To date, metabolomic profiles of FGR/SGA in maternal feces have been examined in only a few studies. Tao et al. investigated the fecal metabolome in women with SGA and AGA fetal growth and identified 23 differential metabolites, including 16 downregulated and 7 upregulated metabolites. Physagulin E, ginkgolide C, and pyrraline were found to be associated with neonatal birth weight. The researchers performed a pathway analysis and found that lipid, amino acid, sphingolipid, fatty acid, and steroid hormone metabolism pathways were enriched in the FGR group (Tao et al., 2023).

Our study is the second to analyze the maternal stool metabolome in SGA fetal growth pregnancies. We compared seven SCFAs and eight AAs in maternal stool samples and found that only one SCFA, hexanoic acid, had a significantly higher concentration in stool samples from women in the SGA group.

SCFAs are produced through the fermentation and decomposition of dietary fiber by anaerobic gut microbiota (Rios-Covian et al., 2020). They play important roles in maintaining gut barrier and modulation of the immune system, among other, by stimulating the secretion of antimicrobial factors and reducing the production of reactive oxygen species and proinflammatory cytokines (Mirmonsef et al., 2012; Bilotta and Cong, 2019; Kim, 2023). They support the development of regulatory T cells (Tregs), which are essential for maintaining immune tolerance. SCFAs can also activate PPAR-γ, a transcription factor involved in suppressing inflammation (Liberato et al., 2012). Additionally, SCFAs bind to free fatty acid receptor 2 (FFAR2) (GPR43) thus regulating the function of the colonic Treg pool (Smith et al., 2013). Notably, SCFAs have been shown to exert strong positive effects on B cells, promoting their activation and differentiation into plasma cells. This function, while beneficial in many contexts, was postulated to contribute in some context to the pathogenesis of autoimmune diseases such as lupus (Kim, 2023).

Microbial metabolic alterations, including changes in hexanoate levels, have also been associated with various pathological conditions. For example, Zhang et al. reported a positive correlation between fecal hexanoate levels and the severity of symptoms in patients with irritable bowel syndrome (IBS), as measured by the IBS Symptom Severity Score (Zhang et al., 2019). De Preter et al. found reduced concentrations of hexanoic acid in patients with Crohn’s disease and ulcerative colitis, and levels of this metabolite negatively correlated with Crohn’s disease activity (De Preter et al., 2015). C5–C8 fatty acids (e.g., pentanoate, hexanoate, heptanoate, and octanoate) were identified as key discriminatory metabolites between healthy individuals and those with inflammatory bowel disease (De Preter et al., 2015). In another study, lower levels of caproic acid were observed in individuals with colorectal polyps compared to controls (Ruiz-Saavedra et al., 2023). Similarly, in people living with HIV, significantly reduced fecal levels of caproic acid were found in those with subclinical atherosclerosis, linked to chronic inflammation, compared to those without (El-Far et al., 2021). In light of these findings, the results of our study appear unexpected, especially considering the commonly reported pro-inflammatory cytokine profile in FGR. However, these observations might contribute to a better understanding of SCFAs’ roles in immune regulation in the future.

Tang et al. investigated gut microbiota metabolism in AGA and FGR pregnancies. The authors identified significant functional changes in metabolites, including methionine, alanine, L-tryptophan, 3-methyl-2-oxovalerate, and ketoleucine. Methionine and alanine were found to be upregulated in FGR cases and were associated with alterations in circulating mRNA expression and microbial abundance (Tang et al., 2024).

The existence of a placental microbiome remains a subject of debate in the literature. As mentioned before, in 2021, Zakis et al. conducted a systematic review of the available published data on the placental microbial composition in healthy pregnancies. The authors identified 24 studies with a low (N=12) to moderate (N=12) risk of bias, which were included in the analysis. A total of 22 studies reported the presence of microorganisms in placental tissues. The most frequently identified genera were *Lactobacillus* (11 studies), *Ureaplasma* (7), *Fusobacterium* (7), *Staphylococcus* (7), *Prevotella* (6), and *Streptococcus* (6) (Zakis et al., 2022). In our study, we investigated the placental microbiome in SGA and AGA groups. We found significantly lower species richness in placental samples collected from the maternal side, whereas species richness was higher in placental samples collected from the fetal side in the SGA group. LINDA-based analyses demonstrated that, at the genus level, the abundances of *Ruminiclostridium 6, Lachnospiraceae A2, Corynebacteriaceae*, and *Faecalibaculum* were significantly lower in placental samples from the SGA group.

Zheng et al. investigated placentas collected from low birth weight and normal birth weight full-term neonates born consecutively at Peking Union Medical College Hospital. They utilized 16S ribosomal DNA amplicon high-throughput sequencing to identify bacteria within placental tissues. The researchers observed significantly lower bacterial richness and evenness in the placentas of low-birth-weight newborns compared to those of normal-weight newborns. *Lactobacillus* was positively associated with birth weight (Zheng et al., 2019). Stupak et al. examined the placental microbiome in pregnancies affected by late FGR. The microbiome was analyzed using LC-ESI-MS/MS mass spectrometry, and bacterial identification was performed through the analysis of bacterial protein sets. Microbiological screening revealed significantly higher relative abundances of pathogenic bacteria (e.g., *E. coli*, *Listeria costaricensis*, and *Clostridiales bacterium*) in placental samples from the FGR group compared to the controls (Stupak et al., 2023). Hu et al. applied 16S sequencing to assess α- and β-diversity and to identify differential taxa features associated with fetal growth. They found a diverse range of flora, predominantly comprising *Proteobacteria, Fusobacteria, Firmicutes*, and *Bacteroidetes*. Neither α- nor β-diversity showed significant differences based on fetal growth status. However, at the taxa level, bacteria associated with a hypoxic environment were observed in women with FGR. A significantly higher prevalence of *Neisseriaceae*, known for its ability to uptake iron-bound host proteins such as hemoglobin, along with an increase in anaerobic bacteria including *Desulfovibrio*, and hydrogen peroxide (H_2_O_2_)-producing *Bifidobacterium* and *Lactobacillus*, reflected the hypoxic status of the placenta in FGR (Hu et al., 2021).

Our study identified a wide range of bacterial taxa in placental samples from both the maternal and fetal sides, with distinct differences observed between AGA and SGA pregnancies. Although our primary objective was to compare microbial composition, it is important to consider the potential functional relevance of these identified bacterial taxa in the context of fetal development and placental function. Emerging evidence suggests that some of the genera identified in our study, such as *Faecalibaculum* and *Lachnospiraceae*, may play roles in regulating immune responses and metabolic processes within the placenta. For example, members of the *Lachnospiraceae* family have been associated with the production of short-chain fatty acids (SCFAs), which can modulate inflammatory pathways and impact immune tolerance (Bui et al., 2025). Similarly, *Faecalibaculum* has been linked to metabolic functions that could potentially influence nutrient transfer between the mother and fetus (Pessa-Morikawa et al., 2022).

Our findings contribute to the ongoing debate regarding the presence and nature of a placental microbiome. Several previous studies have reported the detection of microbial DNA in placental tissues, suggesting a low-biomass but distinct microbial community (Aagaard et al., 2014; Parnell et al., 2017). However, others have challenged these results, attributing microbial signals to contamination or technical artifacts (Leiby et al., 2018; de Goffau et al., 2019). In our dataset, we detected a diverse range of bacterial taxa in placental samples, with differences observed between the maternal and fetal sides, and between SGA and AGA groups. Importantly, our analysis included stringent controls and a comparative assessment with negative controls, allowing us to better distinguish true biological signals from potential contaminants. Several taxa commonly associated with maternal mucosal and cutaneous sites were detected in the placental samples, including *Lactobacillus, Staphylococcus, Escherichia/Shigella, Ureaplasma*, and *Gardnerella*. The presence of *Lactobacillus* aligns with findings from Aagard et al., who reported a non-pathogenic, low-biomass microbiota in term placentas dominated by taxa resembling the oral and vaginal microbiomes (Aagaard et al., 2014). Similarly, *Gardnerella* and *Ureaplasma*—both associated with bacterial vaginosis and intrauterine infections—have been implicated in preterm birth and adverse pregnancy outcomes (Kenfack-Zanguim et al., 2023; Oh et al., 2024). Their presence, particularly in SGA placentas, may suggest a role in altered intrauterine environments contributing to impaired fetal growth. However, the detection of genera such as *Staphylococcus* and *Escherichia/Shigella*—which are frequent contaminants in low-biomass microbiome studies—necessitates cautious interpretation. de Goffau et al. emphasized that many such taxa identified in previous placental studies may arise from reagent or environmental contamination (de Goffau et al., 2019). Our inclusion of AGA controls and relative abundance thresholds helps to mitigate, though not eliminate, this concern. Taken together, these observations support the need for further studies employing metagenomic or culture-based confirmation and rigorous contamination control. The distinct taxonomic signatures observed between SGA and AGA samples, including in both maternal and fetal sides of the placenta, underscore the potential biological relevance of these findings and warrant deeper investigation. Although our results do not resolve the controversy, they support the idea that microbial DNA is detectable in the placenta under certain conditions and may differ by fetal growth status. Further studies with rigorous contamination control and standardized pipelines will be essential to clarify whether these signals represent a resident microbiota or transient microbial DNA exposure.

The strength of our study lies in the homogeneous study group of SGA pregnancies, excluding cases with genetic or major anatomical abnormalities in the fetus or known intrauterine infections causing fetal growth restriction. All potential factors that could influence the maternal microbiome were carefully excluded to minimize possible bias. Only SGA newborns assessed according to the same growth charts were included in the study. Placental samples were collected under sterile conditions and immediately frozen. Stool samples were self-collected by participants following detailed instructions on the collection technique, ensuring no contact with the toilet and using sterile containers. Stool and blood samples were collected within seven days before delivery, allowing for all collections to take place within a short and consistent timeframe. Furthermore, all women delivered between 35 and 41 weeks of gestation, minimizing the impact of potential microbiome changes occurring throughout pregnancy. However, there are several limitations to this study. As a pilot study, the sample size was relatively small. Additionally, we did not collect data on maternal diet and lifestyle. All participants were recruited from the same perinatal center, meaning that potential regional differences in the maternal gut microbiota cannot be entirely ruled out. In our study, we included women with hypertension and pre-eclampsia, conditions that are known to potentially influence the maternal microbiome. However, the prevalence of these conditions in our cohort was low and did not differ significantly between the SGA and AGA groups. Given the small number of affected cases and the lack of significant differences between groups, we did not conduct separate subgroup analyses for these conditions. Nevertheless, we recognize that the presence of hypertension or pre-eclampsia may act as potential confounders in microbiome studies. Despite the limited occurrence of these conditions in our study, it is important to acknowledge their potential impact on the microbiota composition. Future studies with larger sample sizes and more uniform distribution of these conditions would allow for a more detailed assessment of their role in shaping the maternal and placental microbiome in pregnancies complicated by fetal growth restriction.

## 5 Conclusions

Our findings reveal significant differences in microbiota composition and immune interactions between AGA and SGA pregnancies. The altered microbial diversity in placental samples, alongside elevated hexanoic acid levels in maternal stool in the SGA group, suggests a potential link between microbiota and fetal growth. Correlations between specific bacterial taxa and cytokines further support the role of microbiome-immune crosstalk in pregnancy outcomes. Despite being a pilot study, these findings provide valuable insights into the microbiota-immune interplay in pregnancy and lay the groundwork for future research on microbiota-based strategies to support fetal health in at-risk pregnancies.

## Data Availability

The original contributions presented in the study are included in the article/**Supplementary Material**. Further inquiries can be directed to the corresponding author.

## Supplementary material

Supplementary Figure 1 Relative abundance of dominant bacterial taxa in placental samples from SGA and AGA pregnancies. Heatmap showing the relative abundance of bacterial taxa (genus level) that accounted for at least 1% of total reads in at least one placental sample (n = 86). Samples from both the maternal and fetal sides of the placenta are included and are grouped by fetal growth status: small for gestational age (SGA) and appropriate for gestational age (AGA). Each column represents an individual placental sample, and each row corresponds to a bacterial genus. Color intensity reflects relative abundance within each sample. Color intensity reflects relative abundance within each sample. A detection threshold of 500 reads was applied; taxa with fewer than 500 reads in each sample are shown in white.

Supplementary Figure 2 Krona charts of genera with a mean abundance >1% in placental samples from **(A)** SGA and **(B)** AGA groups, comparing maternal and fetal sides of the placenta.
