# Supplementary figures and images for: Maternal and placental microbiome and immune crosstalk in pregnancies with small-for-gestational-age fetuses – a pilot case-control study
Source: Front Cell Infect Microbiol. 2025 Jun 23;15:1596588. doi: 10.3389/fcimb.2025.1596588 (PMC12229884; doi:10.3389/fcimb.2025.1596588)

A

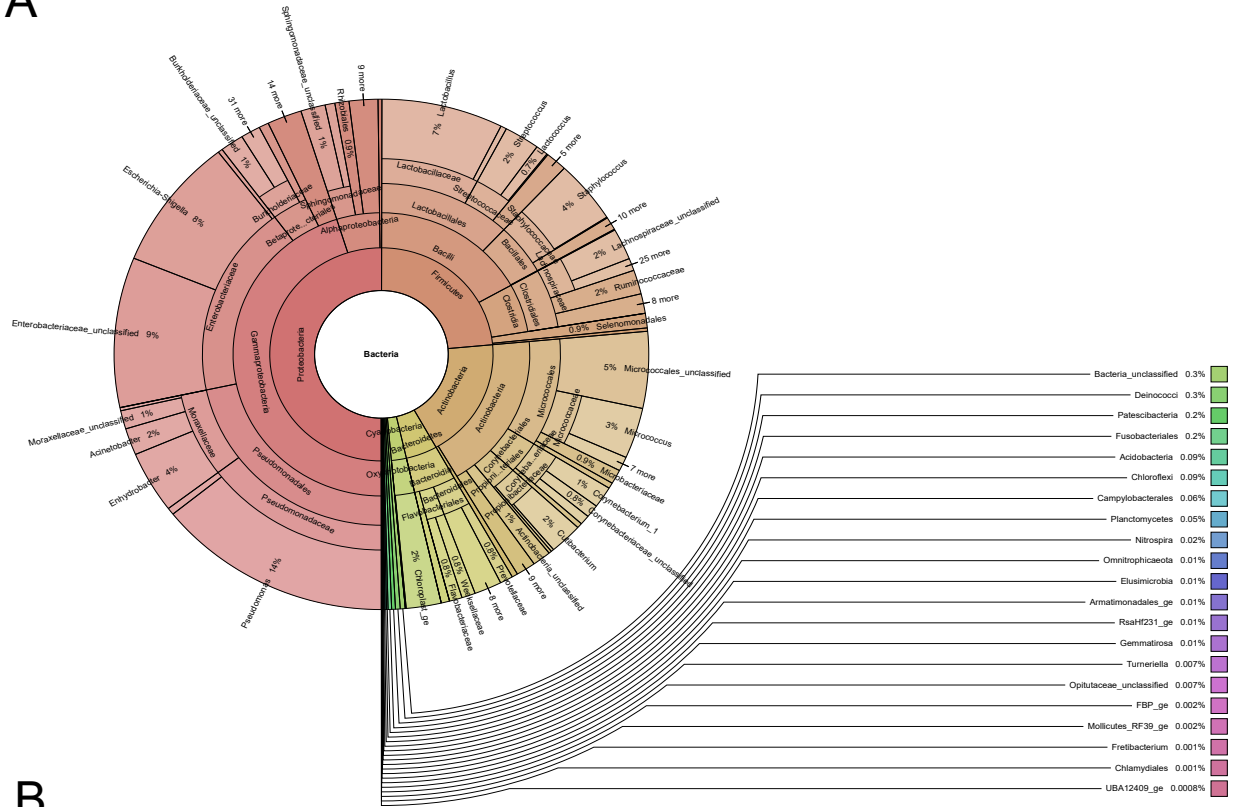

B

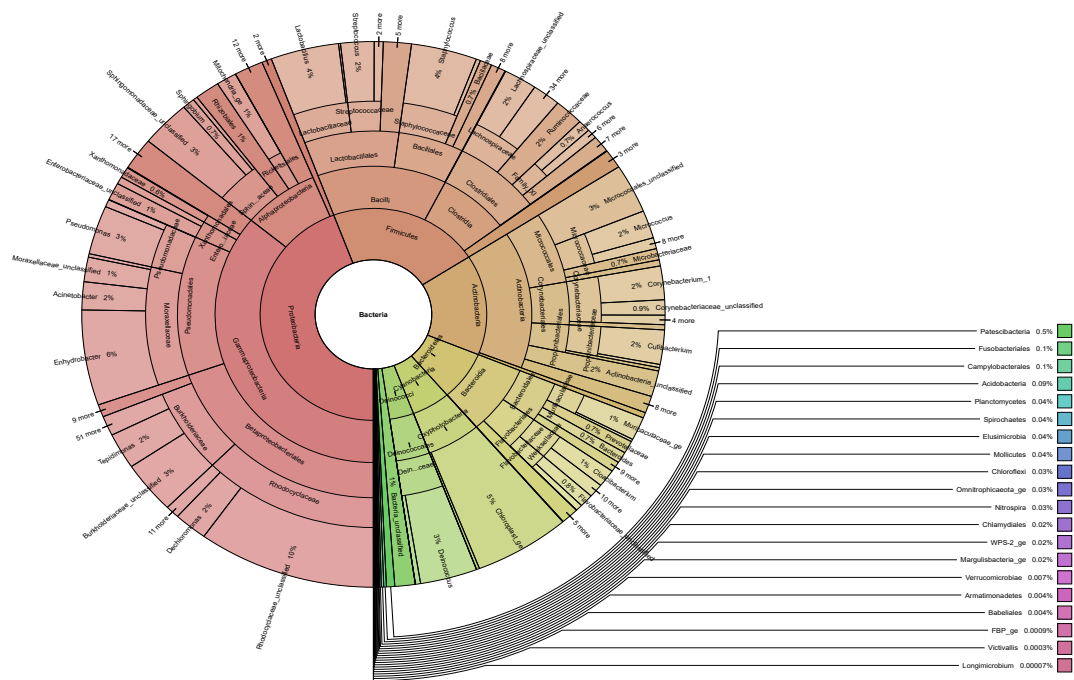

Supplement: Supplementary Figure 1 — Relative abundance of dominant bacterial taxa in placental samples from SGA and AGA pregnancies. Heatmap showing the relative abundance of bacterial taxa (genus level) that accounted for at least 1% of total reads in at least one placental sample (n = 86). Samples from both the maternal and fetal sides of the placenta are included and are grouped by fetal growth status: small for gestational age (SGA) and appropriate for gestational age (AGA). Each column represents an individual placental sample, and each row corresponds to a bacterial genus. Color intensity reflects relative abundance within each sample. Color intensity reflects relative abundance within each sample. A detection threshold of 500 reads was applied; taxa with fewer than 500 reads in each sample are shown in white. [file Image1.pdf]

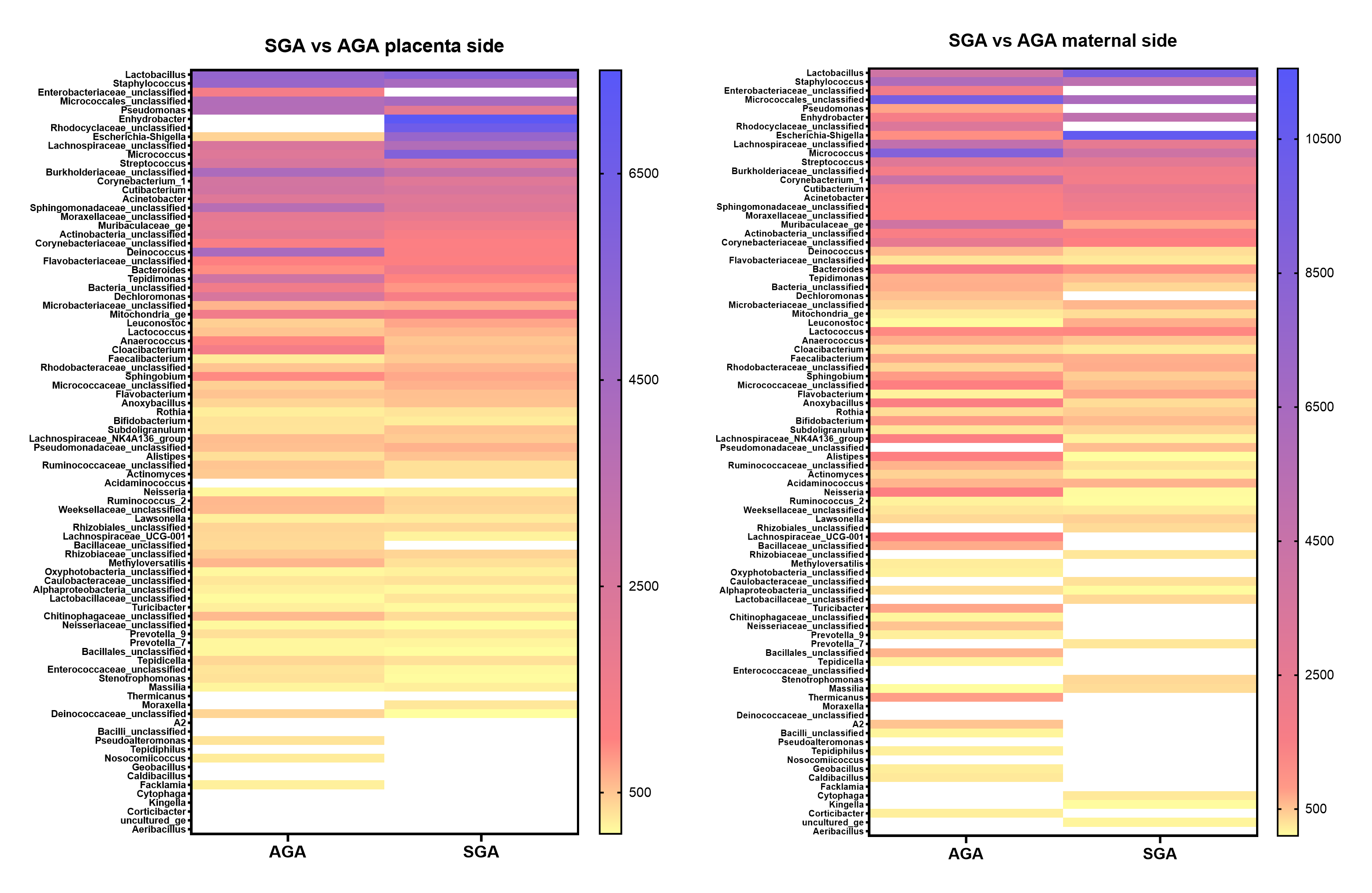

Supplement: Supplementary Figure 2 — Krona charts of genera with a mean abundance >1% in placental samples from (A) SGA and (B) AGA groups, comparing maternal and fetal sides of the placenta. [file Image2.tif]
